# Upward continuation and polynomial trend analysis as a gravity data decomposition, case study at Ziway-Shala basin, central Main Ethiopian rift

**DOI:** 10.1016/j.heliyon.2020.e03292

**Published:** 2020-01-30

**Authors:** Hailemichael Kebede, Abera Alemu, Shimeles Fisseha

**Affiliations:** a School of Earth Science, Addis Ababa University, P.O. Box 1176, Addis Ababa, Ethiopia; b Addis Ababa University Institute of Geophysics, Space Science and Astronomy (IGSSA), Ethiopia

**Keywords:** Geophysics, Geology, Earth sciences, Gravity data separation, Spectral analysis, Vertical derivative, Upward continuation, Trend surface, Surface geology

## Abstract

The first task in quantitative interpretation of a gravity data is separation of the Bouguer anomaly into its regional and residual components which are respectively related to deep and shallow subsurface geology. The decomposition process is subjective and non-unique as there is no single best approach to approximate the low frequency signature. For example, the use of spectral analysis and upward continuation require the wise choice of slope change location and continuation height respectively, which could be chosen differently by different researchers. This requires a need to work on more than one method and select the best to be applied for a given study area. The “best” choice is made based on the anomaly signature of the underlying geology. In this research, the most frequently used methods such as upward continuation and trend surface analysis methods are used and compared to approximate the regional field in Central Main Ethiopian rift bounded between 38^0^00′-39^0^30′E and 7^0^00′-8^0^30′N. The upward continuation height and the order of trend polynomial surface are first chosen, to approximate the regional gravity field signal. Accordingly, an upward continuation height of 6km and first order polynomial trend surface are chosen to be appropriate. Comparison of the two methods shows that the upward continuation technique reflects the shallow source anomalies of the area better than that of the first order linear trend surface. This outcome is verified against the result obtained based on the first vertical derivative method, spectral analysis depth estimation method, well-log data and surface geology of the area. It is therefore recommended to consider the various existing filtering techniques and choose the best candidate for the separation of the regional and residual components of the observed field.

## 1 Introduction

**Figure 1:**
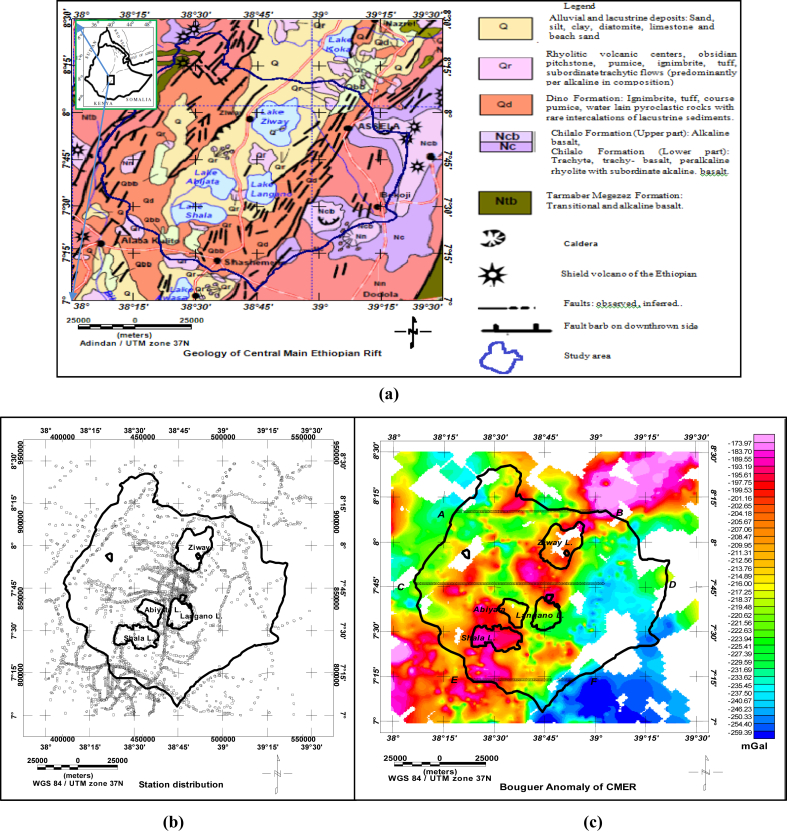
Location and Geology of Ziway-Shala lake basin, Central Main Ethiopian rift (Geo-referenced and digitized from Tefera et al., 1996) (a). Gravity station location distribution (b) and Bouguer Anomaly of the Central Main Ethiopian Rift with three west-east extending profiles (c).

The Ziway-Shala lake basin is located in the central Main Ethiopian rift being bounded within the limit of 38^0^00′-39^0^30′ E and 7^0^00′-8^0^30′ N (Figure 1 (a)).

The main Ethiopian rift (MER) which encompasses Ziway-Shala lakes basin is thought to be developed over a span from the Oligocene to the Quaternary (Woldegabriel et al., 1990). The volcano-tectonic and sedimentation processes associated with this rift system were responsible for geologic and geomorphic features of the region (Le Turdu et al., 1999).

A total of 3012 ground based gravity data obtained from PhD thesis work (Alemu, 1992) and from the Ethiopia Geological Survey (EGS) are considered in this research work. The compiled data set is reprocessed and homogenized with reference to the International Gravity Standardization Network 1971 (IGSN71). The 1967 international gravity formula, a reduction density of 2.67 g/cm^3^ and sea level as a datum are used. The computed complete Bouguer anomaly data are gridded and mapped (Figure 1 (c)) using the Geosoft Oasis montaj version 7.1. It is a known truth that, the computed complete Bouguer anomaly is a superposition of the effect of shallow and deep anomaly signatures which need to be separated in to their respective components before scheduling quantitative interpretation of the data.

The upward continuation technique (Mammo, 2010), polynomial surface fitting (Okiwelu et al., 2010), Wavelet Transform (Xu et al., 2009), spectral analysis (Saibi et al., 2012), graphical methods (Gupta and Ramani, 1980), Preferential filtering (Guo et al., 2013), non-linear filter (Keating et al., 2011) were applied to gravity data to approximate the regional anomaly in their respective study area. These show that no single best chosen method is a reliable to decompose the observed anomaly into its regional and residual components. Each of the methods has a merit and demerit which could be considered depending on the problem and appropriateness. In this research, the most frequently used method such as upward continuation and polynomial trend surface analysis have been applied and compared and the best approach is forwarded for Ziway-Shala lakes basin, Central Main Ethiopian Rift.

## 2 Methods

As stated, among the filtering techniques mentioned in section 1, the upward continuation and trend surface analysis are chosen and their detailed mathematical formulation are given as follow.

### 2.1 The upward continuation

**Figure 2:**
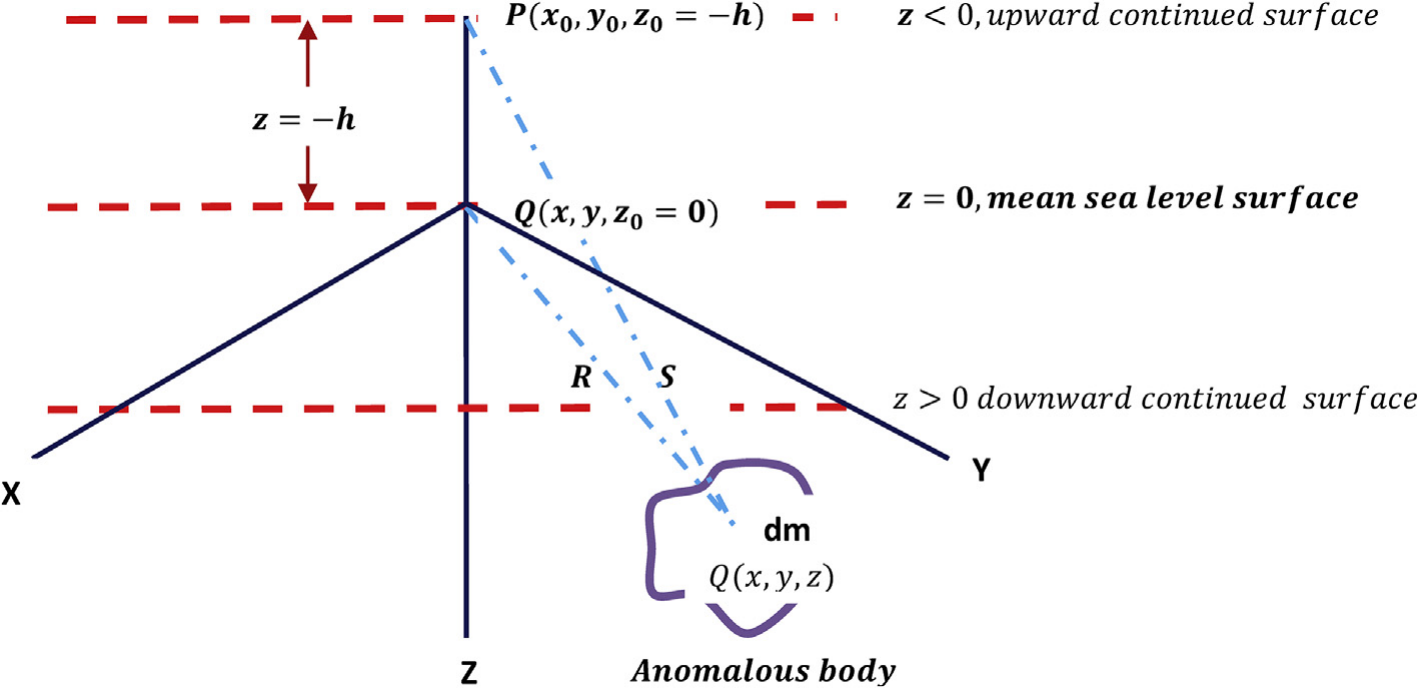
Pictorial representation of upward continuation technique in Cartesian coordinate system.

It is a mathematical technique used to separate the anomaly of the deeper geology from shallower geology (Jacobsen, 1987). It is a transformation of gravity anomaly computed at a point, $Q\left(x_{0},y_{0}\;,z_{0}=0\right),$ on the mean sea level to a point $p\left(x_{0},y_{0}\;,z_{0}=-h\right),$ on some higher flat surface upward continued to, z=−h<0 (Figure 2).

The gravitational attraction per unit mass of an anomalous source body of mass, dm, at mean see level, $O\left(x_{0},y_{0}\;,z_{0}=0\right),$ at distance R from the source location point Q(x,y,z), is computed by

(2.1) $$dg=\frac{Gdm}{R^{2}}=\frac{Gdm}{{{{\left(x-x_{0}\right)}^{2}+\left(y-y_{0}\right)}^{2}+\left(z-z_{0}\right)}^{2}}$$

The vertical component, $dg_{z},$ gives the gravity anomaly,$\;\Delta g_{0},$ of the source at the mean sea level surface point as

(2.2) $$dg_{z}=\Delta g_{0}=\frac{Gdm}{R^{2}}\;\frac{z}{R}=Gdm\frac{z}{R^{3}}=Gdm\frac{z}{{{{{[\left(x-x_{0}\right)}^{2}+\left(y-y_{0}\right)}^{2}+\left(z\right)}^{2}]}^{3/2}}$$

Thus the gravity anomaly,$\;\Delta g_{p},$ of the anomalous source at the upward continued surface point P is

(2.3) $$\Delta g_{p}=\frac{Gdm}{S^{2}}\;\frac{z+h}{S}=Gdm\frac{z+h}{S^{3}}=Gdm\frac{z+h}{{{{{[\left(x-x_{0}\right)}^{2}+\left(y-y_{0}\right)}^{2}+\left(z+h\right)}^{2}]}^{3/2}}$$

Eq. (2.3) represents the gravitational attraction of dm at a height, ‘h’, above mean sea level surface. This filter attenuates the short wavelength anomaly components while enhancing the long wavelength ones. This equation therefore represents an algorithm for developing an upward continuation filter.

### 2.2 Trend surface

It is a mathematical filtering procedure (Menke, 1989) used to approximate the regional component of the gravity field. It is obtained by fitting a low order polynomial surface to the gravity data using least square techniques. Thus, the multiple regression equation involving two independent variables which define the form of the first degree surface fit is given by

(2.4) $$T\left(x_{i},y_{i}\right)=b_{0}+b_{1}x_{i}+b_{2}y_{i}+\varepsilon$$

Where

$$T\left(x_{i},y_{i}\right)-\;is\;estimated\;value\;of\;the\;dependent\;variable$$

$$b_{0}-\;is\;point\;of\;intercept\;on\;the\;y-axis\;T\left(x_{i},y_{i}\right)$$

xandyarethetwoindependentvariablesbeingconsidered

$$b_{1}-\;is\;corrosponding\;change\;in\;T\left(x_{i},y_{i}\right)for\;each\;unit\;change\;in\;x,\;while\;y\;held\;constant$$

$$b_{2}-\;is\;corrosponding\;change\;in\;T\left(x_{i},y_{i}\right)\;for\;each\;unit\;change\;in\;y,\;while\;x\;held\;constant$$

$$\mathrm{Here},\;b_{0\;,\;}b_{1}\;and\;b_{2\;}are\;known\;as\;coefficients\;of\;regression$$

This implies that the regression of the dependent variable on the particular independent variable is measured while holding the value(s) of other variable(s) constant.

From Eq. (2.4) the error ε  is expressed as

$$\varepsilon =\sum {\left(T\left(x_{i},y_{i}\right)-\left(b_{0}+b_{1}x_{i}+b_{2}y_{i}\right)\right)}^{2}\;which\;is\;to\;be\;minimized$$

From concepts of calculus, we have

(2.5) $$\begin{array}{l} \frac{\partial \in }{\partial b_{0}}=-2\sum (T(x_{i},y_{i})-\left(b_{0}+b_{1}x_{i}+b_{2}y_{i}\right)=0\\ \frac{\partial \in }{\partial b_{1}}=-2\sum (T(x_{i},y_{i})-\left(b_{0}+b_{1}x_{i}+b_{2}y_{i}\right)x_{i}=0\\ \frac{\partial \in }{\partial b_{2}}=-2\sum (T(x_{i},y_{i})-\left(b_{0}+b_{1}x_{i}+b_{2}y_{i}\right)y_{i}=0 \end{array}$$

Re-arranging and writing Eq. (2.5) in matrix form, Am=d, we have after some manipulation

(2.6) $$\left(\begin{array}{ccc} n & \sum x_{i} & \sum y\\ \sum x_{i} & \sum {x_{i}}^{2} & \sum x_{i}y\\ \sum y & \sum x_{i}y & \sum y^{2} \end{array}\right)\left[\begin{array}{c} b_{0}\\ b_{1}\\ b_{2} \end{array}\right]\left(\begin{array}{c} \sum T\left(x_{i},y_{i}\right)\\ \sum T\left(x_{i},y_{i}\right)x_{i}\\ \sum T\left(x_{i},y_{i}\right)y \end{array}\right)$$

This is a system of equation with three unknowns and three equations that can be solved as a least square over determined problems given by

(2.7) $${m^{\mathrm{est}}=\left[\begin{array}{c} b_{0}\\ b_{1}\\ b_{2} \end{array}\right]=\left[A^{T}A\right]}^{-1}A^{T}\;\;d$$

Eq. (2.7) is a two-dimensional first degree polynomial surface parameters solution of Eq. (2.4) or (2.6). Similar approach can be followed to get solutions for the second, third and any higher order.

### 2.3 Vertical derivative

The vertical derivative is one of the filtering techniques used for the enhancement of the shallow source features in the data. This transformation magnifies the short wavelength features (Mammo, 2012) which could reflect the residual anomalies. The gridded gravity anomaly data in Figure 1.2(b) can be expressed as a function in Cartesian co-ordinate system, i.e F=f(x,y,z).  The vertical derivative which shows the change of field with depth (z) is expressed as;

(2.8) $$FVD=-\frac{\partial f}{\partial z}$$

The residual anomalies obtained through subtraction of upward continued 6 km and trend surface of order one from Bouguer anomaly are compared against vertical derivative anomaly map to see whether the shallow earth anomalies are satisfactorily picked or not.

### 2.4 Spectral analysis

The profile data extracted from the observed gravity field anomaly (Figure 1 (c)) can further be analyzed by transforming the data from space domain to frequency domain. The Discrete Fourier Transform in one dimension (1D) is given by

(2.9) $$F\left(k\right)=\overset{N-1}{\underset{k=0}{\sum }}f\left(n\right)e^{-i\frac{2\pi kn}{N}}$$

F(k), is the discrete amplitude spectrum which could be respectively written as a sum and product of real and imaginary

F(k)=a(k)+ib(k)

Or

(2.10) $$F\left(k\right)=\left|F\left(k\right)\right|e^{i\varphi \left(k\right)}$$

Where.

$\left|F\left(k\right)\right|=\sqrt{a^{2}\left(k\right)+b^{2}\left(k\right)}$ , is an amplitude spectrum and
k=2πf is The wave number

Spectral analysis thus describes the variation of the energy (amplitude) as a function of frequency (wavelength). The filter estimate subsurface density contrast depth given by Spector and Grant (1970) as

(2.11) $$h=-\frac{1}{4\pi }\left(\frac{logE_{1}-logE_{2}}{K_{1}-K_{2}}\right)$$

Where $E_{1}$and $E_{2\;}$are power spectra of the gravity field.

${\text{logE}}_{1}{\text{ and logE}}_{2}$ are Logarithms of the power spectra
$k_{1}$And $K_{2}$ are wave numbers
h is depth to interfaces (layer boundaries).

Eq. (2.11) gives the average depth, h, which is obtained from difference of the power spectrum curve slopes divided by -4π. This filter designed to determine the top of density contrasts causing gravity anomalies.

## 3 Results and analysis

The Bouguer gravity field (Figure 1(c)) is the cumulative sum of the sources at different depth levels which are broadly classified as sources at shallower and deeper levels. These source anomalies need to be decomposed for better investigation of the subsurface. In this work the upward continuation and trend surfaces approximation methods are applied to accomplish this task. The two methods are particularly chosen because they are used frequently by different researchers and because of their simplicity in understanding how they work. However, one method shouldn't be simply picked and applied on approximation of low frequency signature. That is, one needs to work on whether the chosen regional approximation method best represent the deep geology which in turn should result in shallow earth (residual anomaly) of the area than the other methods. This paper is devoted to select a single appropriate method for Bouguer gravity separation.

### 3.1 Upward continuatio

**Figure 3:**
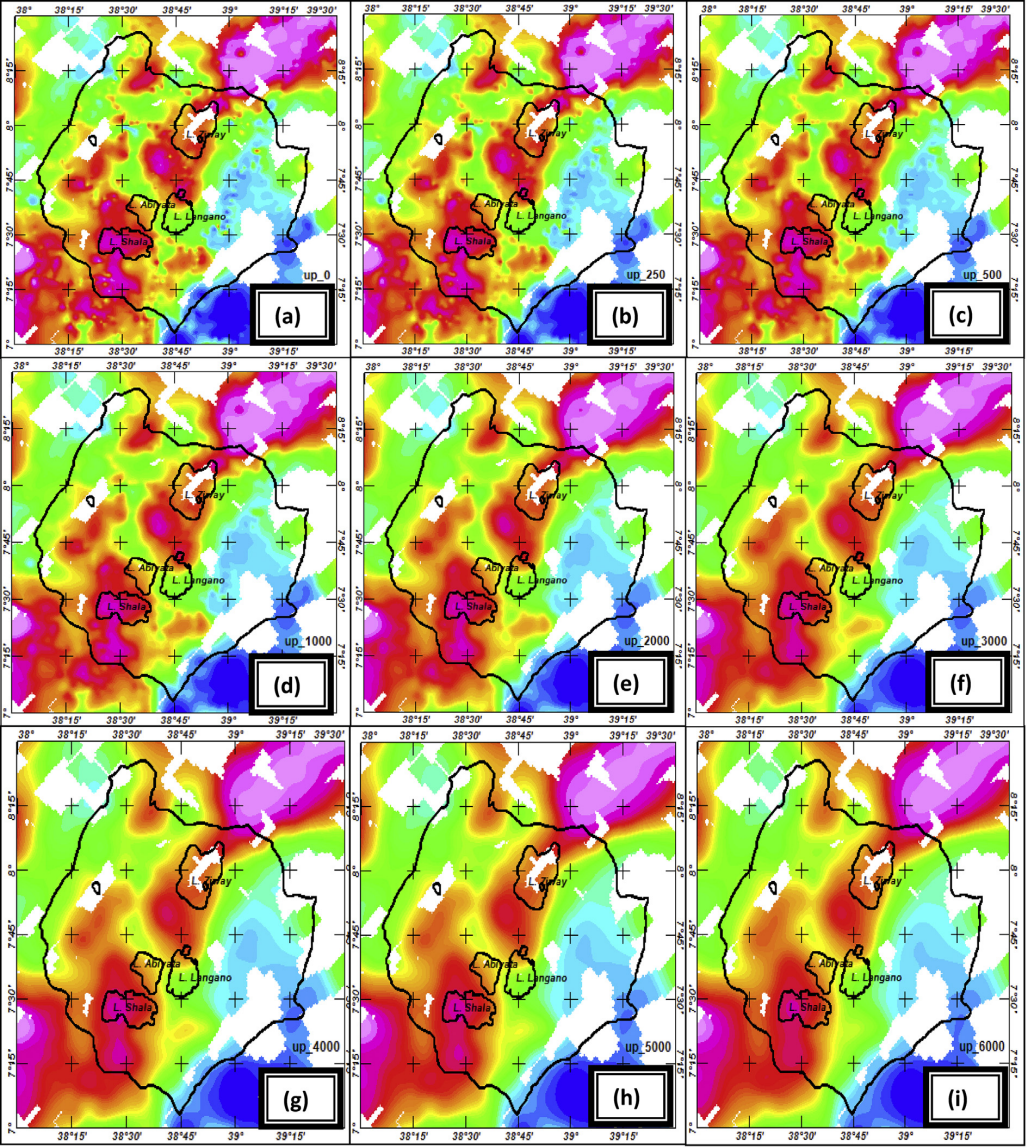
Bouguer anomaly (a) upward continued to height of 250m (b) to height of 500m (c) to height of 1.0 km (d) to height of 2km (e) to height of 3km (f) to height of 4km (g) to height of 5km (h) and to height of 6km (i) to image sources buried at and below the depths 125m (b) 250m (c) 500m (d) 1.0 km (e) 1.5 km (f) 2.0 km (g) 2.5 km (h) and 3.0 km (i) respectively.

**Figure 4:**
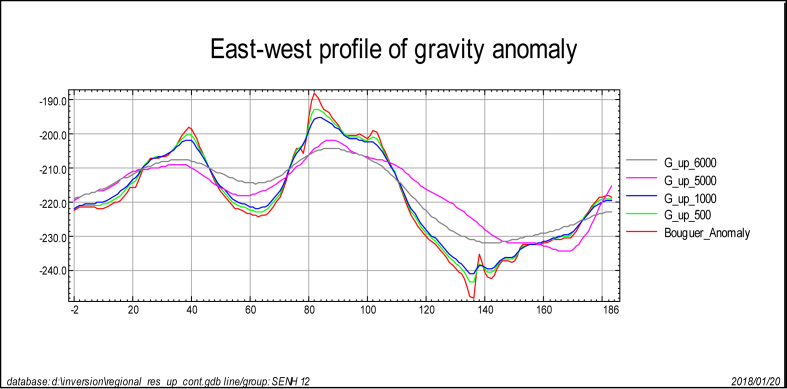
Regional gravity anomaly profiles through upward continuation to height of 500m, 1km, 5km and 6km across west-east at **7**^**0**^**45′N** latitude. The gray color profile anomaly map (upward continued to 6km) is smoother than others anomaly maps.

To estimate the appropriate upward continuation height the Bouguer anomaly (Figure 1 (c)) are upward continued to a height of 250 m, 500 m, 1.0 km, 2 km, 3 km, 4 km, 5 km and 6 km. As the continuation height increases the effect of shallower features disappears (Figure 3(b-i)). Particularly, at a continuation height of 6000 m (Figure 3(i)) most of the shorter wavelength are removed. This is evidently justified by the west-east extending Bouguer gravity anomaly profile (Figure 4) subjected to upward continuations. The anomaly profile curve gets smoother as continuation heights increased from 500m to 1000m, from 1000m to 5000m and from 5000m to 6000m. Thus, the continuation height chosen characterizes the regional anomaly field in the region under study.

According to Jacobsen (1987) when the gravity field is upward continued to a height z, it maps the sources found at and below the depth,z/2. Thus, the upward continuation of Bouguer anomaly (Figure 3(a)) to height of 250m (Figure 3(b)) to height of 500m (Figure 3(c)) to height of 1.0 km (Figure 3(d)) to height of 2.0 km (Figure 3(e)) to height of 3.0 km(Figure 3(f)) to height of 4.0 km (Figure 3(g)) to height of 5.0 km (Figure 3(h)) and to height of 6km (Figure 3(i)) are used to image sources buried at and below the depths 125m (b), 250m(c), 500m(d), 1km(e), 1.5km(f), 2km(g), 2.5km(h) and 3 km(i) respectively.

**Figure 5:**
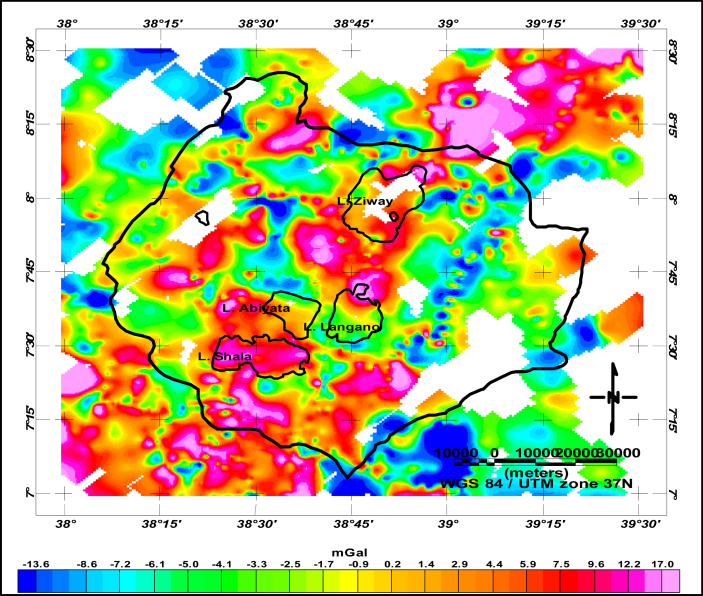
Residual anomaly map of Ziway-Shala lakes basin after the anomaly sources deeper than 3km (upward continued 6km) are removed which is characterized by negative and positive gravity values.

The residual anomaly obtained after subtracting the regional field estimated through up continuation to a height of 6000m is shown in Figure 5.

### 3.2 Trend surface analysis method

**Figure 6:**
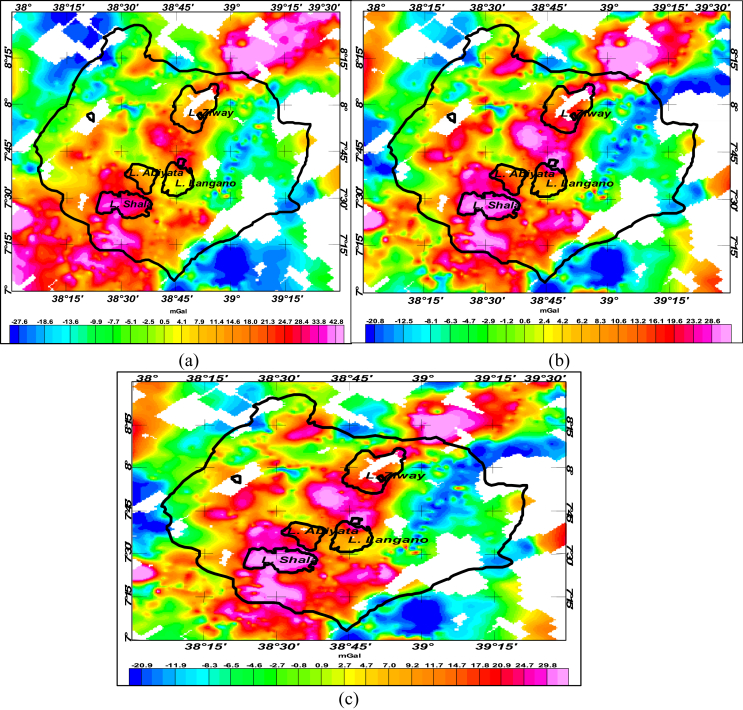
Linear first, second and third order polynomial trend surface removed from Bouguer anomaly generates the residual anomalies (a), (b) and (c) respectively.

The residual anomalies obtained through approximation of regional field with trend surface of first, second and third orders respectively are shown in the Figure 6(a, b and c). These anomalies don't show the varied residual anomalies of the area as that of the upward continuation method.

**Figure 7:**
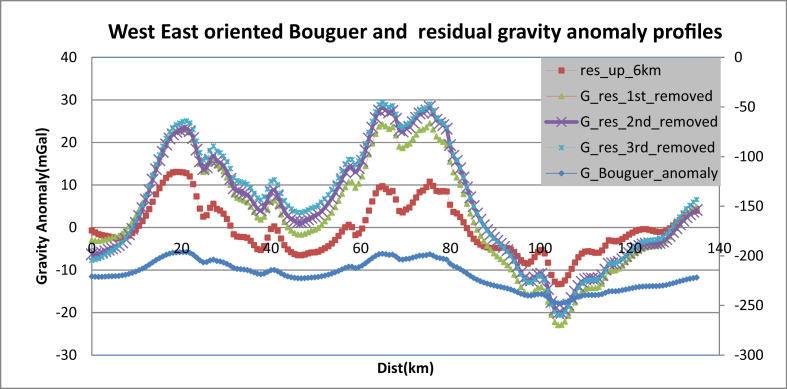
Relationship between Bouguer anomaly and residual anomalies generated by subtraction of regional anomalies estimated through upward continuation and trend surface fits of first, second and third order polynomials.

The correlation coefficient between the west-east (along **7**^**0**^**45′N** latitude) oriented residual anomaly profiles obtained using upward continuation to height of 6km with those of first, second and third order trend surface polynomial are calculated to be 0.91, 0.87 and 0.86 respectively. Figure 7 supports consistency of the computed correlation coefficients for the upward continuation and trend surface methods. Moreover, the correlation coefficients 0.91 corresponding to the first order polynomial trend surface best elucidate the shallow geologic features of the area than those of the second and third order surface trend polynomials.

### 3.3 Vertical derivative

**Figure 8:**
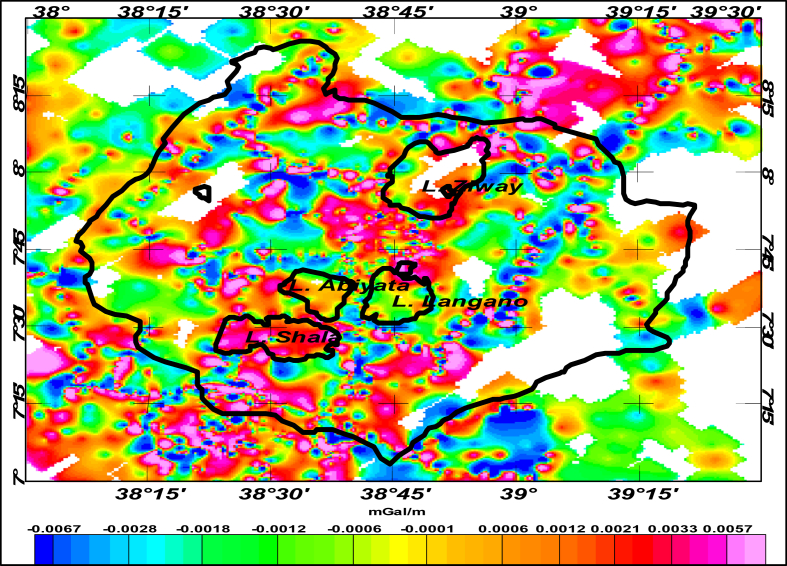
First vertical derivative of Bouguer anomaly.

To validate the residual field results obtained from upward continuation to a height of 6km, the first vertical derivative of the Bouguer anomaly is generated (Figure 8). The results appear to better match the residual anomalies generated using upward continuation technique than the one generated through first order trend polynomial fit. The comparison is based on residual (or local) anomaly signatures resulting from shallow origin subsurface masses. Shallower source signatures are better observed using the residual obtained through upward continuation than the residuals using trend surface analysis.

### 3.4 Spectral analysis

Spectral analysis is one of the quantitative depth estimation methods for gravity anomaly source bodies. The upward continuation method is another filtering technique which is used to determine anomaly sources occurring at variable depths. These two techniques can be jointly applied to estimate source depths and make a comparison of the results obtained from the two methods.

**Figure 9:**
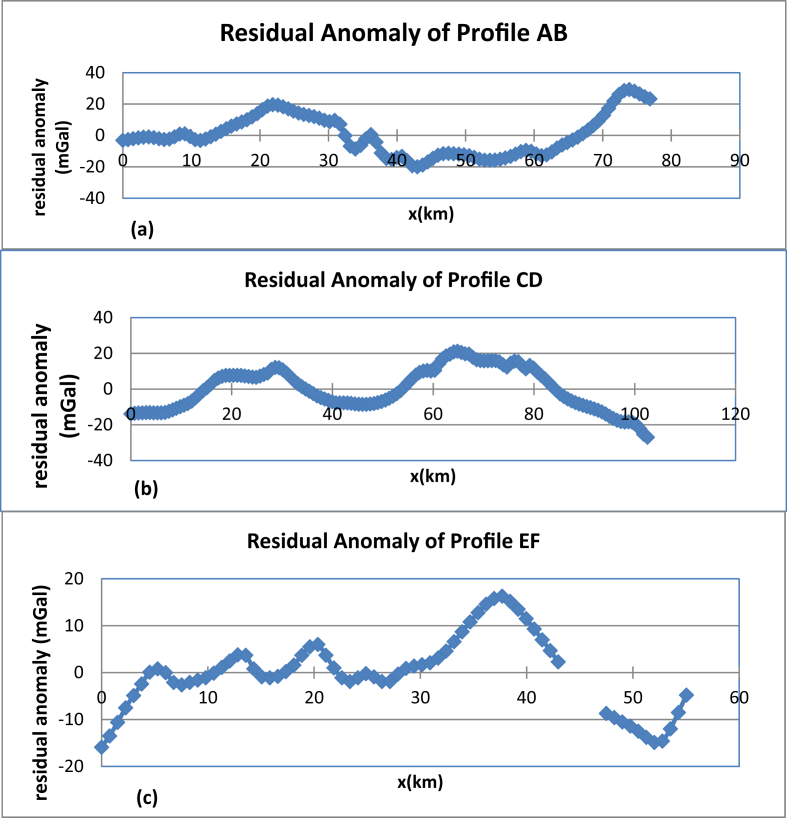
Residual anomaly maps along profile AB (a), along profile CD (b) and along profile EF (c).

**Figure 10:**
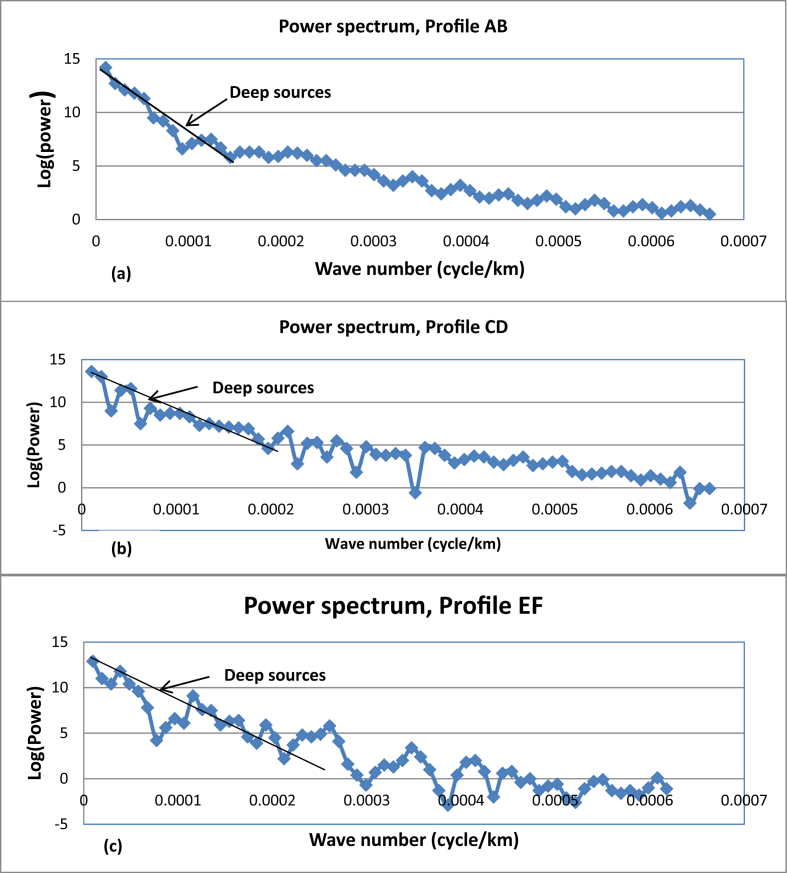
Logarithmic power spectrums of profiles AB (a), CD (b) and EF (c).

**Figure 11:**
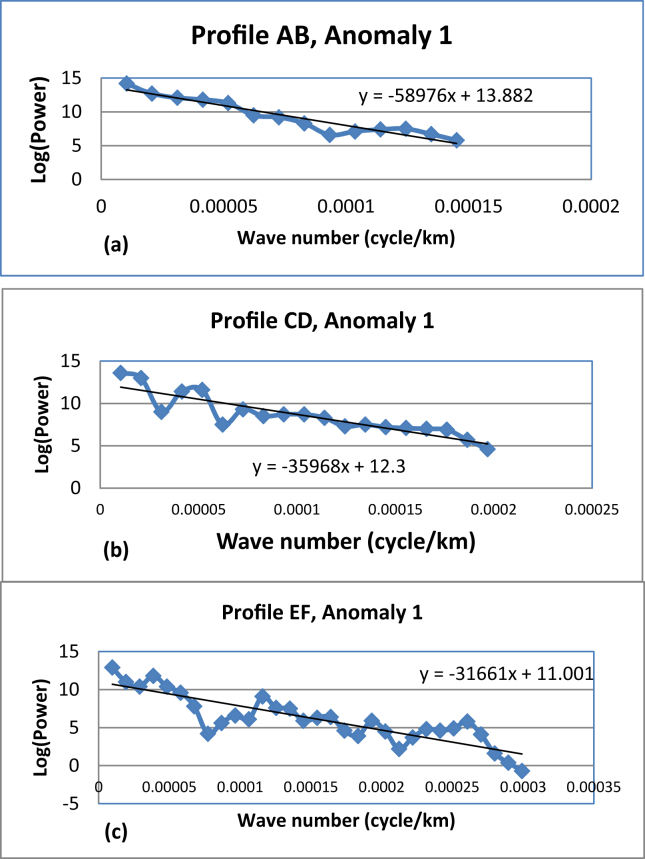
Lowest wave number anomalies signal of profile AB (a), CD (b) and EF(c) with their trend polynomial line fit which could help to read the gradient estimates.

In this study, profiles AB, CD and EF are extracted from Bouguer anomaly map (Figure 1 (c)) and a first order polynomial is removed to generate the residual anomalies (Figure 9(a), (b) and (c)). The residual anomaly field is analyzed by transforming the data from space domain to discrete frequency domain (Equation 2.9). The one dimensional Fourier series expansion of these profiles data are subjected to spectral analysis to decompose anomaly signals based on wavelength. The average depth to the anomaly sources contrast can be estimated based on the slope of power spectral magnitude versus wave number. Figure 10(a), (b) and (c) shows the three logarithmic amplitude spectrums of profile AB, CD and EF with their corresponding lowest wave number anomalous signal (Figure 11(a), (b) and (c)).

Though we were able to extract the deep, the intermediate and shallow depth density contrast locations from logarithmic power spectrum profile maps (Figure 10); we are interested in the lowest frequency signal signature (Figure 11). This lowest frequency signal corresponds to the deep source density contrast depth.

The mean depth values corresponding to the deeper density contrast for the three selected profiles AB (north), CD (central) and EF (south) approximated using spectral method are 4695.54 m, 2636.95 m and 2520.78 m respectively. The average value of the estimated depths amounts to 3.284 km (Table 1). This mean depth possibly approximates the deeper source (crystalline basement) in the region considered.

**Table 1:** The regional gravity anomaly source fields and approximated depth of the three profiles AB, CD and EF.

Profile Name	Gradients	Depth(m)	Source Location	Average(m)
(Profile AB)	58976.00	**4695.54**	**Deep**	3284.423
(Profile CD)	35968.00	**2636.95**	**Deep**	
(profile EF)	31661.00	**2520.78**	**Deep**	

The mean depth (3.284km) estimated using spectral analysis fairly agrees with the top of density contrast causing the regional gravity anomalies estimated using upward continuation filter (3km).

### 3.5 Comparison with well log data and surface geology

The analysis made in sections 3.1, 3.2, 3.3 would help to conclude that the upward continuation at 6.0 km height better approximates the regional field anomaly of the area than the other continuation heights. This is supported by the well-log data geologic model of the Aluto-Langano Geothermal field (Hutchison et al., 2016; Wilks et al., 2017; Abera and Mizunaga, 2018). The model shows the depth to crystalline basement rock is found at depth below 2.5km. This basement rock is the source of the regional gravity anomaly deduced by the upward continuation height of 6 km. According to Jacobsen (1987) when the gravity field is upward continued to a height 6 km, it maps the sources found at and below the depth 3 km.

**Figure 12:**
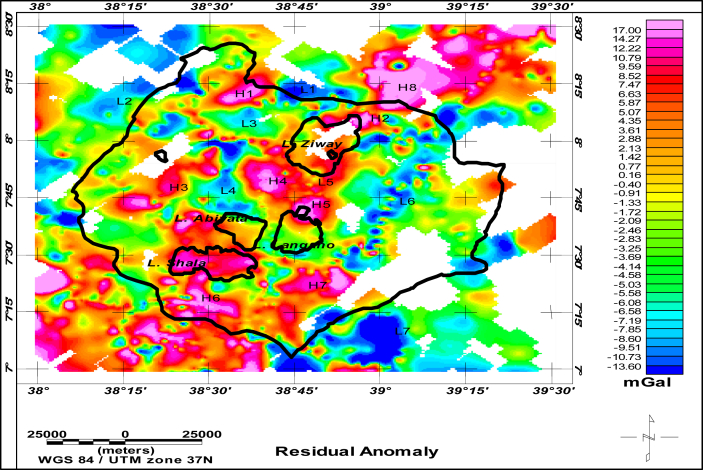
Residual anomaly of the region categorized as high (H) and low (L) anomalies.

The residual anomaly map (Figure 12) with color contrast designated by “H” for high and “L” for low gravity anomaly values are compared with surface geology of the area constructed by (Tefera et al., 1996; Molin and Corti, 2015). The contrasting gravity anomalies and there corresponding geologic sources are summarized in Table 2. The comparison made is consistent.

**Table 2:** Relationships of the anomalous source with surface Geology of the area.

S.N	Signal Strength	Correlation with existing geological map	Sources
1	H1	Nazret pyroclastic rocks: Rhyolitic ignimbrite lava flows and domes (Late Miocene –Pliocene) embedded in Chefe Donsa Pyroclastic deposits (Rhyolitic ash flows and fall deposits) Dino formation: Ignimbrite, tuff, course pumice, water lain pyroclastic rocks with rare intercalations of lacustrine sediments	(Molin and Corti, 2015) (Tefera et al., 1996)
2	H2	Southern extension of Bora-Bericha rhyolites: Rhyolitic and trachytic lava flows and domes, pyroclastic deposites(Pleistocene-Holocene) Rhyolitic volcanic centers, Obsidian pitchstone, pumice, ignimbrite, tuff, subordinate trachytic flows (predominantly per alkaline in composition)	(Molin and Corti, 2015) (Tefera et al., 1996)
3	H3	Wonji basalts: Basaltic lava flows, scoria and phreatomagmatic deposit(Pleistocene-Holocene) overexposed on Chefe Donsa Pyroclastic deposits (Rhyolitic ash flows and fall deposits)	(Molin and Corti, 2015)
4	H4, H5 and H8	Bora-Bericha rhyolites: Rhyolitic and trachytic lava flows and domes, pyroclastic deposites(Pleistocene-Holocene) Rhyolitic volcanic centers, Obsidian pitchstone, pumice, ignimbrite, tuff, subordinate trachytic flows (predominantly per alkaline in composition)	(Tefera et al., 1996) Molin and Corti (2015)
6	H6	Wonji basalts: Basaltic lava flows, scoria and phreatomagmatic deposit(Pleistocene-Holocene)	(Molin and Corti, 2015)
7	H7	Chilalo volcanics(formation): Trachytic lava flows(alkaline basalt) and pyroclastic deposits (Pliocence-Pleistocence)	(Molin and Corti, 2015) (Tefera et al., 1996)
9	L1	Alluvial and Lacustrine sediments: sand, silt, clay, diatomite lime stone and beach sand (Pleistocence-Holocene)	(Tefera et al., 1996) (Molin and Corti, 2015)
10	L2	Nazret pyroclastic rocks: Rhyolitic ignimbrite lava flows and domes (Late Miocene –Pliocene) embedded in Chefe Donsa Pyroclastic deposits (Rhyolitic ash flows and fall deposits)	Molin and Corti (2015)
12	L3	Chefe Donsa Pyroclastic deposits (Rhyolitic ash flows and fall deposits) Alluvial and Lacustrine sediments: sand, silt, clay, diatomite lime stone and beach sand (Pleistocence-Holocene)	(Tefera et al., 1996) (Molin and Corti, 2015)
13	L4	Chefe Donsa Pyroclastic deposits (Rhyolitic ash flows and fall deposits) Alluvial and Lacustrine sediments: sand, silt, clay, diatomite lime stone and beach sand (Pleistocence-Holocene)	(Tefera et al., 1996) (Molin and Corti, 2015)
14	L5	Accumulated sediments	(Searle and Gouin, 1972)
15	L6	Nazret pyroclastic rocks: Rhyolitic ignimbrite lava flows and domes (Late Miocene –Pliocene)	(Molin and Corti, 2015)
16	L7	Chefe Donsa Pyroclastic deposits (Rhyolitic ash flows and fall deposits)	(Molin and Corti, 2015)

## 4 Conclusion

The Bouguer gravity anomaly need to be decomposed into its regional and residual component sources before making any interpretation. Though, numerous decomposition techniques are available in literature for approximating the low frequency signals, no specific information is available for the choice of a particular representative method. Furthermore, the method of separation is non-unique and thus no single technique exists. For example, the use of spectral analysis and upward continuation require the wise choice of slope change locations and continuation heights respectively. This requires to work on more than one method and select the best for a given study area. In this research, upward continuation and trend surface analysis methods are compared to map shallow surface geologic and tectonic features of the study area. Accordingly, the upward continuation technique is chosen to be the more reliable approach as it mapped the shallow earth gravity sources better than the trend surface analysis. This is confirmed by comparison of the obtained anomaly with the anomaly generated through analysis of first vertical derivative, power spectral analysis, well-log data and surface geology of the area. The subjectivity and non-uniqueness of the existing separation techniques thus requires comparison of the various decomposition techniques in order to choose the best method for the approximation of the regional and the residual fields of a given area.

## Declarations

### Author contribution statement

H. Kebede: Conceived and designed the experiments; Performed the experiments; Analyzed and interpreted the data; Contributed reagents, materials, analysis tools or data; Wrote the paper.

A. Alemu: Analyzed and interpreted the data; Contributed reagents, materials, analysis tools or data; Wrote the paper.

S. Fisseha: Analyzed and interpreted the data.

### Funding statement

This research did not receive any specific grant from funding agencies in the public, commercial, or not-for-profit sectors.

### Competing interest statement

The authors declare no conflict of interest.

### Additional information

No additional information is available for this paper.
